# Decreased HDL-C Levels as a Predictor of Organ Failure in Acute Pancreatitis in the Emergency Department

**DOI:** 10.3390/life13071602

**Published:** 2023-07-21

**Authors:** Ana Rocío Venegas-Tamayo, Olga Mariel Peña-Veites, Martha Alicia Hernández-González, Cornelio Barrientos-Alvarado

**Affiliations:** 1 High Specialty Medical Unit No. 1, National Medical Center of Bajío, Mexican Social Security Institute, Leon 37320, Guanajuato, Mexico; 2 Department of Physiology, Higher School of Medicine, National Polytechnic Institute, Mexico City 11340, Mexico

**Keywords:** high-density lipoprotein cholesterol, acute pancreatitis, multiple organ failure, Atlanta classification, Bedside Index for Severity of Acute Pancreatitis, modified Marshall score

## Abstract

High-density lipoprotein cholesterol (HDL-C) is reported as a biomarker of systemic inflammation and multi-organ failure (MOF), which has been rarely investigated in acute pancreatitis (AP), a frequent condition in the emergency department (ED). The objective was to study the predictive capacity of the decrease in HDL-C to the progression of MOF in AP in the ED; analyzing 114 patients with AP for one year in a longitudinal and prospective study, AP severity was obtained by the Atlanta classification, in relation to modified Marshall and Bedside Index for Severity in Acute Pancreatitis (BISAP) scores, and clinical and laboratory parameters in a 48 h hospital stay. The area under the receiver operating characteristic (ROC) curve was used to estimate the validity of the predictor and define optimal cut-off points. It was found that AP was classified as severe in 24.5%, mainly for biliary etiology (78.9%) and female sex (73.6%). As a biomarker, HDL-C decreased from 31.6 to 29.5 mg/dL in a 48 h stay (*p* < 0.001), correlating negatively with the increase in severity index > 2 and the modified Marshall (*p* < 0.032) and BISAP (*p* < 0.009) scores, finding an area under the ROC curve with a predictive capacity of 0.756 (95% CI, 0.614–0.898; *p* < 0.004) and a cut-off point of 28.5 mg/dL (sensitivity: 79%, specificity: 78%), demonstrating that the decrease in HDL-C levels serves as a useful indicator with a predictive capacity for MOF in mild to severe AP, during a 48 h hospital stay in the ED.

## 1. Introduction

Acute pancreatitis (AP) is one of the most common gastrointestinal disorders requiring hospitalization [1]; it is an important and frequent condition in the emergency department (ED) and can cause death for non-timely medical care [2]. AP in Mexico is the 17th cause of mortality, comprising 0.5% of deaths, and the fifth cause of admission to the ED for adults with biliary and alcoholic etiologies [3].

AP is caused by tissue inflammation pancreatic and the consequent release of enzymes, mainly trypsinogen to trypsin, causing its self-digestion and the powerful stimulation of macrophages that induce the production of proinflammatory cytokines [1]. AP constitutes the final common pathway of multiple etiopathogenic stimuli and can trigger local necroinflammatory changes [4], multisystemic effects, and compromise organs, with structural lesions and systemic inflammatory process, affecting nearby tissues in addition to pancreatic tissue, producing organ failure [5] with damage to the pancreatic parenchyma and basal lipase elevation, with typical clinical characteristics: pain, radiation, and changes by imaging methods, associated with the presence of reactive oxygen species [6]. Early identification of patients who are prone to developing severe AP is becoming important, which would help determine close surveillance or aggressive intervention by physicians in the ED [7].

Recent studies show that patients with severe AP can alter serum lipid levels and are often accompanied by changes in cholesterol levels [8]. It has been reported that cholesterol flux can regulate inflammation and the response of monocytes, macrophages, and lymphocyte expansion [9], suggesting remodeling and dysfunction of proteins that modify high-density lipoprotein cholesterol (HDL-C) levels by raising low-density cholesterol (LDL-C) and oxidative stress, contributing to endothelial dysfunction and the inflammatory process in pancreatitis [10]. Thus, the assessment of altered cholesterol levels is proposed as a predictor of organ failure in AP [11], reported that low HDL-C levels are associated with a considerable risk of persistent organ failure in AP [12].

HDL-C also has anti-inflammatory, antioxidant, antiaggregatory, anticoagulant, and profibrinolytic properties, and the decreased HDL-C may be an independent indicator by which the patient progresses to multi-organ failure (MOF) [13], pancreatic necrosis, or even death, where measurement of HDL-C levels may predict organ failure in AP [14]. There is no evidence to support the relationship between HDL-C and the incidence of MOF in the ED.

The Atlanta classification divides the severity of AP into mild, moderately severe, and severe based on the presence of local or systemic organ failure (transient or persistent): mild severity courses without organ failure and without local complications or systemic; moderate severity courses with transient organ failure (resolved in less than 48 h) and/or with local or systemic complications without persistent organizational failure; and severe presents organ failure (<48 h), either from a single organ or multiorgan [15], which makes it possible to classify the morphology of this disease and to predict its clinical severity by applying imaging severity indices, depending on the presence or absence of necrosis. Interstitial edematous AP is the most frequent and diffuse non-necrotizing inflammation that occurs, and necrotizing AP is generally affected by the pancreatic gland, pancreatic tissue, or both [16], suggesting that a modified Marshall score with a score >2 for organ failure [15] indicates a course in relation to: PaO2/FiO2, creatinine, blood pressure, being persistent when the score >2 for organic failure exceeds 48 h of stay hospital and transitory if it is lower [17]. The modified Marshall score has been reported with predictive ability indicating a course of transient organ failure [18].

On the other hand, the Bedside Index for Severity of Acute Pancreatitis (BISAP) with a score >2 is suggested to predict mortality and as an early indicator of organ failure in AP, with reassessment at 24 and 48 h of hospital stay [19]. BISAP is more convenient to use with fewer items, being evaluated with five criteria: BUN >25 mg/dL, age >60 years, presence of pleural effusion, Glasgow neurological deterioration <15, and systemic inflammatory response syndrome (SIRS). The latter includes two or more of the following criteria: temperature <36 °C or >38 °C, respiratory rate >20/min or PaCO_2_ < 32 mmHg, heart rate >90/min, leukocytes <4000 or >12,000 cells/mm^3^ or reticulocytes >10% [20]. The BISAP clinical scoring system has been reported to show diagnostic accuracy in predicting severe AP, according to the Atlanta classification [21]. Our objective was to evaluate as a clinical parameter the predictive capacity of the decrease in HDL-C levels in the progression to MOF in AP during 48 h of hospitalization in the ED.

## 2. Materials and Methods

### 2.1. Patient Selection

A study was carried out with the following design: analytical, observational, longitudinal and prospective. A total of 114 patients admitted to the ED of the High Specialty Medical Unit Number 1, in León Guanajuato, for one year (May: 2019–2020) presented AP with the following criteria: >18 years, either gender, transient organ failure <48 h, and possible deterioration from previous concomitant diseases or local complications such as acute peripancreatic fluid collections, pseudocysts, acute necrotic collections, and necrosis. We excluded patients with chronic pancreatitis induced by trauma, with lipid-lowering treatment, who died within the study time.

### 2.2. Ethical Approval

This study was carried out in accordance with the guidelines of the Declaration of Helsinki of 1975 (2013 revision) and was approved by the Hospital’s research ethics committee, folio: R-2019-1001-039 SIRELCIS in May 2019; High Specialty Medical Unit No. 1 Bajío National Medical Center, Mexican Social Security Institute, with the informed consent of the patient.

### 2.3. Patient Information and Data Collection

Patient charts were reviewed for information on demographics (age, gender, and etiology), medical history, smoking, alcohol use, pre-existing comorbidities such as diabetes mellitus, obesity, and pre-existing organ dysfunction (including chronic respiratory disease, renal, and cardiovascular disease); the etiology and the time of evolution of the AP of the studied patients were obtained.

### 2.4. Classification and Definitions: Atlanta Classification, Modified Marshall and BISAP Scores

AP was defined by compatible abdominal pain, elevated lipase >150 U/L (ELITech Clinical Systems LIPASE SL, Mexico City, Mexico), and imaging study using abdominal ultrasound or computed tomography. The clinical and morphological severity was established by the Atlanta classification [15,16] according to the following criteria: mild AP is defined as no organ failure or systemic or local complications, while moderately severe AP as one or more transient organ failure or systemic or local complications, and severe AP consists of persistent organ failure for more than 48 h. Organ failure was detected through a modified Marshall score ≥ 2 for at least one of the three organs involved (cardiovascular failure, respiratory failure, and renal failure); the final points of the modified Marshall scores were calculated by summing the points for each of the three organs [17,18,22], and early risk of organ complication was determined using a BISAP score >2 points [17,18,21] upon admission and 48 h after hospitalization by trained physicians and experienced radiologists.

### 2.5. Laboratory Investigations

Clinical laboratory studies were performed: complete blood count, blood chemistry, and complete lipid profile, including HDL-C, LDL-C, very low-density cholesterol (VLDL-C), total cholesterol (TC), triglycerides (TGs), nitrogen blood urea (BUN), lipase, hematocrit (Hto), C-reactive protein (CRP), calcium, and creatinine. Blood samples were collected within the first 2 and 48 h after hospitalization in the ED; they were immediately spun in a refrigerated centrifuge, obtaining the serum, and then frozen at −80 °C for analysis using a blood analyzer automated clinical chemistry (Biossays 240 Plus, Snibe Co. Diagnostic, Shenzhen, China).

### 2.6. Statistical Analysis

In this study, the biomarkers, ratios, and multifactorial scores were evaluated upon admission and at 48 h of the symptom onset.

The descriptive analysis was performed for the qualitative variables with the distribution of frequencies and percentages of the number of patients in relation to the variables: sex, etiology, severity by Atlanta classification and modified Marshall and BISAP scores.

For quantitative variables, based on the age, severity by Atlanta classification, the modified Marshall and BISAP scores, and paraclinical studies (CT, TGs, BUN, lipase, CRP, Hto, VLDL-C, LDL-C, HDL-C, calcium, and creatinine), the normal distribution was evaluated with the Kolmogorov–Smirnov test, whether the continuous data showed a normal distribution, the continuous values were expressed by means ($\bar{x}$) ± standard error (SE). The two-way analysis of variance test was used to analyze the differences in the means, assuming the normal distribution of the data sets for the modified Marshall and BISAP scores in relation to the Atlanta classification and the paraclinical studies in relation to the modified Marshall and BISAP scores. Paired samples *t*-test was used to evaluate the mean differences of the paraclinical values at admission and 48 h after hospitalization. Pearson’s correlation test was performed to assess the relationships of modified Marshall and BISAP scores between levels of paraclinical studies.

The area under the receiver operating characteristic (ROC) curve, that is, AUC was used to evaluate the performance of predictions; ROC curves were obtained using the modified Marshall and BISAP scores, and the HDL-C, calcium, and creatinine levels to assess the predictive capacity of organ failure. A variable with an AUC above 0.7 was considered useful, while an AUC between 0.8 and 0.9 indicated excellent diagnostic accuracy [23].

The IBM SPSS Statistics version 22 program was used. The probability α was 0.05.

## 3. Results

### 3.1. Epidemiological Characteristics and Severity Classification of AP

For the present study, according to the census of 114 patients with AP admitted to the ED, the average age was 49.1 years, with a predominance of 73% for the female gender and 78% for biliary etiology (Table 1). We found a severe Atlanta classification of 24%, increasing to 28% at 48 h of hospital stay (Table 2); the modified Marshall score > 2 was 29%, decreasing to 22% at 48 h of hospital stay, and the BISAP score > 2 was 14%, which increased to 21% at 48 h of hospital stay (Table 3).

### 3.2. Modified Marshall and BISAP Scores

In Table 4, using the modified Marshall and BISAP scores as predictors of organ failure in relation to the severity by Atlanta classification (mild and severe), a significant difference was shown in the average modified Marshall and BISAP scores > 2 upon admission (*p* < 0.001) and at 48 h (*p* < 0.001) of hospital stay in the severe Atlanta classification with respect to the mild Atlanta classification; however, it is noteworthy that for the BISAP score there was a significant increase (*p* < 0.05) in admission to the ED with respect to 48 h of hospitalization for the severe Atlanta classification.

### 3.3. Paraclinical Studies

Paraclinical studies showed higher TGs, BUN, lipase, CRP, and creatinine levels upon admission to the ED and lower Hto, LVDL-C, HDL-C, and calcium levels compared to normal ranges, observing a decrease in HDL-C and calcium levels at 48 h of hospital stay (*p* < 0.001) and an increase in creatinine levels at 48 h of hospital stay (*p* < 0.01) (Table 5).

### 3.4. Correlation Analysis

The Pearson’s correlations between the modified Marshall score and the paraclinical variables upon admission to the ED were positive with respect to BUN (*p* < 0.001) and creatinine (*p* < 0.001) and negative for Hto (*p* < 0.002) and HDL-C (*p* < 0.002). For the BISAP score upon admission to the ED, they were positive for BUN (*p* < 0.001) and creatinine (*p* < 0.001), and negative for Hto (*p* < 0.002) and HDL-C (*p* < 0.009), maintaining the negative correlation for HDL-C at 48 h of hospital stay (*p* < 0.005), showing a tendency for HDL-C to decrease in the face of an increase in the BISAP score in patients with a prognosis of organ failure. On the other hand, creatinine levels showed a positive correlation at admission and at 48 h of hospitalization for both scores studied (*p* < 0.001), being an acceptable indicator of an increase in relation to the increase in the score of the modified Marshall and BISAP scores as predictors of organ failure; for calcium, the correlations were negative, although they were not significant (Table 6).

### 3.5. Receiver Operational Characteristic Analysis

**Figure 1:**
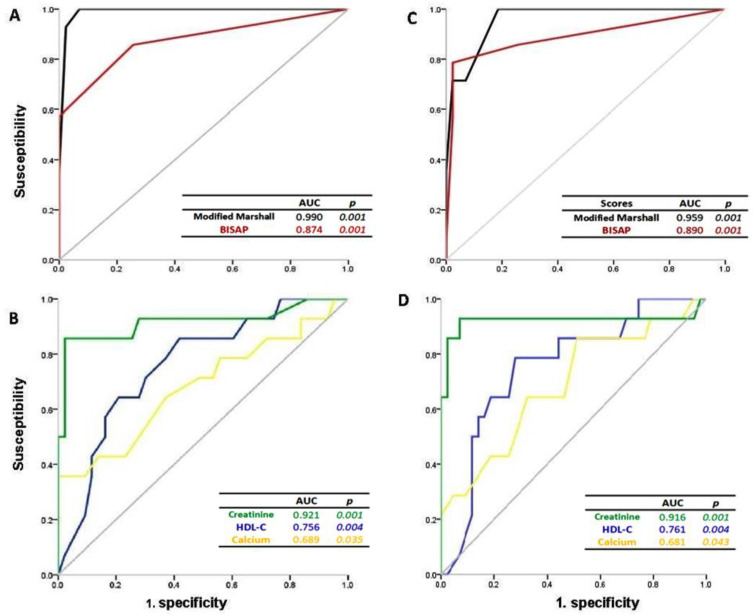
Receiver operating characteristic curves as a prediction of organ failure in patients with AP: (**A**) dependent on the modified Marshall and BISAP scores; (**B**) dependent on the HDL-C, calcium, and creatinine levels at admission to the ED; (**C**,**D**) after 48 h of hospital stay (n = 114), *p* < 0.05.

Analysis of the ROC curves upon admission to the ED to assess the prediction of organ failure using the modified Marshall and BISAP scores showed an area under the curve (AUC) of 0.990 (95% CI, 0.970–1.000; *p* < 0.001) and 0.874 (95% CI, 0.748–1.000; *p* < 0.001), respectively (Figure 1A).

HDL-C levels, as a useful indicator of organ failure, showed an AUC of 0.756 (95% CI, 0.614–0.898; *p* < 0.004) with a cut-off point of 28.5 mg/dL as predictive capacity with a sensitivity of 79% and a specificity of 72%, on the other hand, calcium levels showed an AUC of 0.689 (95% CI, 0.523–0.856; *p* < 0.035) with a cut-off point of 8.35 mg/dL with a sensitivity of 86% and a specificity of 49%; and creatinine levels showed an AUC of 0.921 (95% CI, 0.791–1.000; *p* < 0.001) with a cut-off point of 1.95 mg/dL with a sensitivity of 86% and a specificity of 98% (Figure 1B).

These same factors, analyzed after 48 h of hospital stay, showed an AUC for Marshall scores of 0.959 (95% CI, 0.914–1.000; *p* < 0.001) and for BISAP scores of 0.890 (95% CI, 0.764–1.000; *p* < 0.001) (Figure 1C).

HDL-C levels as a useful indicator of organ failure showed an AUC of 0.761 (95% CI, 0.622–0.900; *p* < 0.004) with a cut-off point of 28.5 mg/dL as predictive capacity with a sensitivity of 86% and a specificity of 58%; on the other hand calcium levels showed an AUC of 0.681 (95% CI, 0.503–0.860; *p* < 0.043) with a cut-off point of 7.00 mg/dL with a sensitivity of 65% and a specificity of 100% and creatinine levels showed an AUC of 0.916 (95% CI, 0.804–1.000; *p* < 0.001) with a cut-off point of 1.85 mg/dL with a sensitivity of 86% and a specificity of 98% (Figure 1D).

### 3.6. ANOVA Analysis between HDL-C Levels vs. BISAP Score

**Figure 2:**
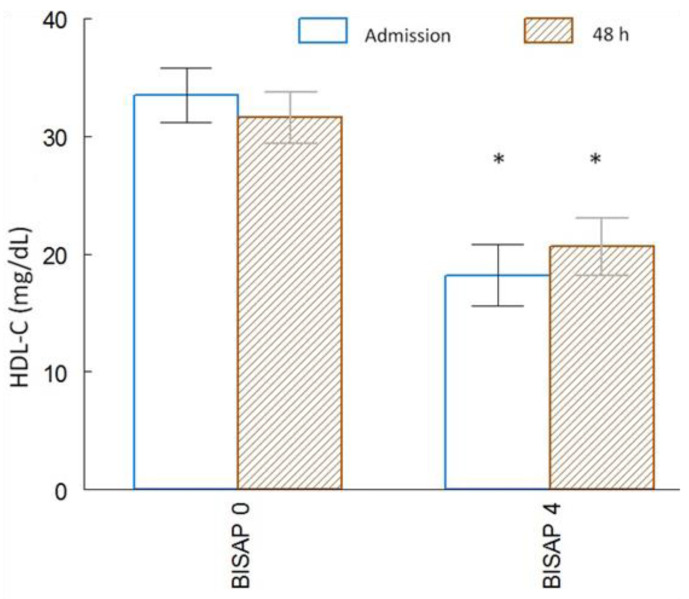
HDL-C levels at admission and 48 h of hospitalization of patients with AP associated with the BISAP score (n = 114); bifactorial ANOVA, * *p* < 0.05.

Figure 2 shows a significant decrease in HDL-C levels in relation to the BISAP score upon admission to the ED were 33.32 ± 2.30 mg/dL for BISAP score 0 and 18.2 ± 2.59 mg/dL for the BISAP 4 score (*p* < 0.01); at 48 h of hospital stay HDL-C levels were 31.51 ± 2.19 mg/dL for BISAP score 0 and 20.62 ± 2.44 mg/dL for BISAP score 4 (*p* < 0.01), corroborating that with a higher BISAP score, there are lower HDL-C levels.

## 4. Discussion

The severity of AP can be predicted based on clinical, laboratory, and radiologic risk factors, several severity score systems, and biomarkers. Some of these can be performed upon admission to assist in the triage of patients, while others can only be obtained during the first 48 [24]. AP, as one of the leading causes of ED care in the world, does not have a biochemical marker that can predict MOF, despite several staging scales, severity grading systems, and serum markers to aid in the diagnosis, selection of patients, and timely treatment, which can be used within 48 h after hospitalization [25]. The results found in the present study suggest that in the ED, in patients with AP, a decrease in the level of HDL-C is a simple and independent biomarker that can provide predictive information for MOF.

In this study, an incidence of AP like in previous studies was seen, in which the female gender is the most affected, and in which it is reported that the main etiology of AP is the biliary cause, with an average age of 40 years (Table 1) [26], although its etiology is complex and not known for certain, the two most common causes are gallstones and alcohol [15]. Females are more likely to have biliary-related pancreatitis [27]; the increase in the incidence of AP has been mostly seen in women aged < 35 and men between the ages of 35 and 54 [28].

Likewise, it was observed that the incidence of severity obtained by the Atlanta classification does not revert after 48 h in this study, reaching 28% of the patients studied (Table 2). Most patients with AP have a mild course, while 10–20% of them develop significant severe disease [29]; the pathological stage was reported with an overall mortality of 3–6%, which increases to 30% in severe AP [30], justified by the progression of the acute inflammatory response to SIRS and/or multiorgan failure [31]. On the other hand, the BISAP score showed an increase of 7% (Table 3), the latter being a tool with the possibility of predicting the development of MOF that incorporates SIRS criteria [32]; as reported with a frequency of 15–20% mild to severe progression in AP, it as an easy score that is calculated from data available in the first 24 h after admission [33].

As is known, the higher the staging of the Atlanta classification, the higher the Marshall and BISAP scores found [32,33]; however, in this study, only the BISAP score showed an increase after 48 h of hospitalization (Table 4), which is consistent with a possible prediction of MOF [34], as a simple and inexpensive method, based on vital signs, age, laboratory tests, neurological status, and images; thus, the BISAP score as a useful scale > 2 predicts systemic complications, this feature is extremely significant given that the first 24 to 48 h are the most crucial and decisive time window in the management of AP [35], several studies have validated the performance of the BISAP score as a predictive tool for AP severity; the diagnosis of organ failure was based on a modified Marshall score, and a score of 2 or more was considered to be the presence [36]; therefore, the modified Marshall and BISAP scores in this study showed predictive information for MOF; showing acceptable AUC between 0.75 and 0.9 for the ROC test for both scales (Figure 1A,C), as predictors of MOF, regarding the multifactorial prognostic scores, the modified Marshall and BISAP scores were evaluated, due to their potential use in a clinical setting and the studies published with their assessment on the prognosis of AP severity [15]. The modified Marshall score caught a lot of attention given its simplicity and applicability in staging AP depending on the degree and duration of organ failure, and that is what has been adopted by the revised Atlanta classification published in 2012 and widely used since. According to the revised Atlanta classification, organ failure is determined by the modified Marshall score; thus, the determination of the severity of AP is one of the most important first steps in the management of AP. It helps in selecting appropriate treatments, ensuring proper patient triage, initiation of applicable therapies, and stratifying patient risk for complications [37]. The definition of the duration of organ failure is important: if it resolves within 48 h, it is called transient organ failure; if it persists for more than 48 h, it is called persistent organ failure; and when organ failure affects more than one organ it is called MOF [15].

In addition to recognized scoring systems, recent studies have focused on simple biomarkers that could provide predictive information. Among the paraclinical factors associated with AP in this study (Table 5), hypertriglyceridemia was found, which has been reported as an associated factor with a frequency of 12% to 39% [38], the elevation of BUN >20 mg/dL at 24 h of hospitalization has been associated with a mortality between 6% and 20% [38]; likewise, it has been reported that the BUN at 24 h is a risk factor for significant results such as multiple organ failure in severe AP [39], the increase in serum lipase in terms of prognostic assessment of severity in AP with a sensitivity of 90–100% and a specificity of 99% and the increase in CRP with a sensitivity of 80% and a specificity of 60%, with a predictive value of 86% [40]. It has been reported that the assessment of the severity of acute pancreatitis using indicators such as CRP has been shown to be useful in predicting AP [41]. In this study, the increase in BUN levels (Table 5) as an indicator was shown to have a positive relationship with the increase in modified Marshall (*p* < 0.001) and BISAP (*p* < 0.001) scores (Table 6).

On the other hand, the low Hto levels found (Table 5) and the negative relationship with the increase in the modified Marshall and BISAP scores (Table 6) are reported as an evaluation factor in the first 24 h, mentioning it as an indicator of risk of developing necrosis, associated with lower creatinine clearance, related to mortality [36]. Hematocrit has been described in several studies as a possible marker of severity in acute pancreatitis [42].

The calcium levels shown in this study, as previously reported, are a useful factor for the prognosis of severity and mortality in AP [43]. For this study, the decrease in calcium levels did not show an AUC greater than 0.75 in the ROC diagnosis, finding a cut-off point <8.5 mg/dL (Figure 1B,D), which decreased after 48 h of hospitalization (Table 5), this decrease was related to the increase in the modified Marshall score (Table 6). Calcium is a valuable tool for a rapid assessment of MOF in patients with AP [44], although calcium levels have been reported to have a higher predictive value than severity scores such as SIRS and Ranson [45].

Regarding serum creatinine levels, it has been reported that levels >1.8 mg/dL are indicators of developing pancreatic necrosis in 93% [46], a situation found in the present study in which serum creatinine levels were >2 mg/dL during the 48 h of hospitalization, with an AUC of the ROC tests >0.9 and a cut-off point >1.8 mg/dL (Figure 1B,D). Previous studies have reported that early changes in creatinine levels in AP are useful indicators of disease severity and mortality, demonstrating that creatinine levels within 48 h of admission are a highly accurate predictor of necrosis pancreatic, which are closely associated with the severity of AP [46].

In this study, the participation of the lipid parameters TGs, TC, HDL-C, and LDL-C with the incidence of hospital MOF in patients with AP in the ED was examined. Additionally, a decrease in HDL-C levels as a possible predictor of MOF in AP was studied [47]. A significant decrease of 29.57 mg/dL was observed during the 48 h stay in the ED in relation to normal values of 40–60 mg/dL (*p* <0.001) (Table 5). We demonstrated that HDL-C levels upon admission and during the 48 h hospital stay were significantly lower. Data were related to an increase in severity by modified Marshall (*p* < 0.032) and BISAP (*p* < 0.009) scores, a relationship that was maintained at 48 h of hospital stay for the BISAP score (*p* < 0.005) (Table 6).

Hyperlipidemia is the third most common cause of AP; the presence of hypertriglyceridemia was related to severe AP and was associated with local and systemic complications [48,49]. Previous studies show that patients with severe acute pancreatitis are often accompanied by hypocholesterolemia [12,50]. Regarding the lipid profile status in acute pancreatitis, a decrease in HDL level has been reported; an abnormal lipid profile may have a role in the pathogenesis of acute pancreatitis in which HDL-associated antioxidant defense is impaired [51]. In this study, it was shown that the decrease in HDL-C levels is a clinical indicator with possible predictive capacity, with a comparable diagnosis in the analysis of significant ROC curves (*p* < 0.004) and an AUC > 0.750 with a cut-off point of 28.5 mg/dL for MOF, which was maintained from admission to 48 h of hospital stay in the ED (Figure 1B,D). AUC showed superior prognostic performance in predicting severe outcomes; this decrease in HDL-C levels also showed a significant relationship with the increase in severity by BISAP score from 0 to 4 both upon admission and at 48 h of hospitalization (Figure 2) (*p* > 0.05), with the decrease in HDL-C levels being an indicator of the incidence of progression to MOF in relation to the severity of AP in the ED. Results were consistent with studies that report HDL-C as an independent prognostic factor of progression to MOF in the AP [50,52], where HDL-C at normal levels and its mimetics can have antioxidant and anti-inflammatory functions by affecting local systems, being related as an inhibitor of the inflammatory response secondary to reactive oxygen species. Therefore, the decrease in HDL-C levels can lead to a severe systemic inflammatory response, where patients with SIRS progress to organ failure [53,54,55]; likewise, the deficiency and alteration in HDL-C functions can present changes and reduce the protective properties of HDL-C levels, decreased HDL-C levels are an independent prognostic factor for adverse outcomes for MOF [56]. The activity of serum paraoxonase (the lipophilic antioxidant component of high-density lipoprotein cholesterol) decreased in patients with acute pancreatitis. In this regard, it was hypothesized that impaired high-density lipoprotein-associated antioxidant defense may contribute to the severity of the disease [57]. It has recently been published that high-density lipoprotein cholesterol levels are effective in the early prediction of MOF in patients with AP [58,59]. HDL-C, as a useful parameter in combination with several clinical parameters, can improve the predictive sensitivity of AP, being able to form part of a multivariate model for AP prediction [60]. Several studies have shown that the first 48 h after the symptom onset is very important to identify those patients at risk of developing complications [61]. This early assessment of the severity of AP becomes crucial, especially in the first 48 h since this period is considered important to define the therapeutic approach.

Showing the following relevance in the present study, it is one of the first detailed studies of the impact of HDL-C as a predictor useful of MOF in patients with AP in ED; likewise, multivariate adjustments were used for predictors of severity and MOF in particular calcium and creatinine in the analysis of results, and patients with AP without any medical treatment were included since lipid-lowering treatments have been reported to be associated with a lower risk of pancreatitis [62]. In addition, studies in animal models suggest that treatment with lipid-lowering agents may be beneficial in both acute pancreatitis and chronic pancreatitis [63,64], so this study is part of the studies that provide valuable information on the predictive capacity of reducing HDL-C levels in AP.

Recognizing some limitations, since it was a prospective and observational study, it had a limited number of patients, and patients with lipid-lowering treatment were not included, so it is recommended that future studies increase the number of patients and analyze patients with lipid-lowering treatment.

## 5. Conclusions

The results of this study showed for the first time that decreased HDL-C levels are related to increased modified Marshall and BISAP scores and demonstrated potential as a possible useful indicator with a predictive ability for MOF in mild to severe AP over 48 h of hospitalization in the ED. As a useful parameter, HDL-C levels, in combination with several clinical parameters, form part of a multivariate model for AP prediction, being an additional tool to stratify patients at risk of AP and its application in the ED, which can improve clinical care and management strategies in AP in the ED.

## Figures and Tables

**Table 1:** Frequencies of socio-epidemiological data in patients with AP at admission and 48 h of hospitalization in the ED (n = 114).

Variables	$\bar{x}$ (SE)	Min–Max
Age	49.17 (2.37)	22–99
		N. Patients (%)
Sex	Male	30 (26.3)
	Female	84 (73.7)
Etiology	Biliary	90 (78.9)
	Alcoholic	6 (5.3)
	Medications	6 (5.3)
	Hypertriglyceridemia	6 (5.3)
	Uremia	4 (3.5)
	Post-operative	2 (1.7)
	Total	114 (100)

**$\bar{x}$**—mean; SE—standard error.

**Table 2:** Frequencies of severity stratification by Atlanta classification in patients with AP at admission and 48 h of hospitalization in the ED (n = 114).

Atlanta Classification	N. Patients (%)	
	Admission	48 h
Mild	84(73.7)	82(71.9)
Moderate severe	2(1.7)	0(0)
Severe	28(24.6)	32(28.1)
Total	114(100)	114(100)

**Table 3:** Frequencies of stratification modified Marshall and BISAP scores in patients with AP at admission and 48 h of hospitalization in the ED (n = 114).

	Score	N. Patients (%)	
		Admission	48 h
Modified Marshall	<2	80 (70.2)	88 (77.2)
	>2	34 (29.8)	26 (22.8)
	Total	114 (100)	114 (100)
BISAP	<2	98 (85.9)	90 (78.9)
	>2	16 (14.1)	24 (21.1)
	Total	114 (100)	114(100)

BISAP—Bedside Index for Severity of Acute Pancreatitis.

**Table 4:** Average modified Marshall and BISAP scores in patients with mild and severe AP according to the Atlanta classification, at admission and 48 h of hospital stay (n = 114), the mean ± SE is expressed, bifactorial ANOVA, *p* < 0.05.

Atlanta Classification								
	Mild				Severe			
	Admission		48 h		Admission		48 h	
Scores	$\bar{x}$	(SE)	$\bar{x}$	(SE)	$\bar{x}$	(SE)	$\bar{x}$	(SE)
Modified Marshall	0.38	0.14	0.28	0.11	4.0 *	0.26	3.5 *	0.61
BISAP	0.24	0.06	0.24	0.06	2.29 *	0.16	2.88 *^,α^	0.42

* *p* < 0.05 vs. mild; ^α^ *p* < 0.05 vs. admission on severe Atlanta classification; $\bar{x}$—mean; SE—standard error; BISAP—Bedside Index for Severity of Acute Pancreatitis; ANOVA—analysis of variance.

**Table 5:** Average results of paraclinical studies of patients with AP, at admission and 48 h of hospital stay (n = 114), the mean ± SE is expressed; paired samples *t*-test, *p* < 0.05.

	Admission	48 h
Paraclinical Studies	$\bar{x}$ (SE)	$\bar{x}$ (SE)
TGs (mg/dL)	439.52 ± 147.91	441.28 ± 130.89
TC (mg/dL)	183.99 ± 19.29	190.65 ± 25.7
BUN (mg/dL)	30.68 ± 3.96	33.45 ± 7.67
Lipase (U/L)	7211.31 ± 1568.14	7856.98 ± 1876.89
CRP (mg/dL)	37.75 ± 3.27	35.87 ± 5.35
Hto (%)	38.35 ± 1.63	34.67 ± 2.78
VLDL-C (mg/dL)	93.94 ± 29.4	80.83 ± 24.5
LDL–C (mg/dL)	79.73 ± 7.16	86.89 ± 9.52
HDL-C (mg/dL)	31.68 ± 1.71	29.57 ± 1.60 *
Calcium (mg/dL)	8.41 ± 0.11	8.05 ± 0.11 *
Creatinine (mg/dL)	2.15 ± 0.47	2.37 ± 0.46 *

* *p* < 0.001 vs. admission; $\bar{x}$—mean; SE—standard error; HDL-C—high-density lipoprotein cholesterol; LDL-C—low-density lipoprotein cholesterol; VLDL-C—very low-density lipoprotein cholesterol—TC: total cholesterol—TGs—triglyceride; BUN—blood urea nitrogen: Hto—hematocrit; CRP—C-reactive protein.

**Table 6:** Shows the significant Pearson’s correlations between the modified Marshall and BISAP scores vs. paraclinical studies levels at admission and 48 h of hospitalization (n = 114), *p* < 0.05.

	Admission				48 h			
	Scores							
	Modified Marshall		BISAP		Modified Marshall		BISAP	
Paraclinical Studies	r	(*p*)	r	(*p*)	r	(*p*)	r	(*p*)
BUN	0.655	(0.001)	0.636	(0.001)	0.643	(0.001)	0.698	(0.001)
Hto	−0.451	(0.002)	−0.402	(0.003)	−0.401	(0.002)	−0.409	(0.002)
HDL-C	−0.285	(0.032)	−0.345	(0.01)	−0.221	(0.098)	−0.365	(0.017)
calcium	−0.217	(0.067)	−0.228	(0.076)	−0.235	(0.061)	−0.276	(0.089)
creatinine	0.641	(0.001)	0.601	(0.001)	0.598	(0.001)	0.586	(0.001)

BISAP—Bedside Index for Severity of Acute Pancreatitis; BUN—blood urea nitrogen: Hto—hematocrit; HDL-C—high-density lipoprotein cholesterol.

## Data Availability

The data presented in this study are available within this article.
